# Fully Printed Cellulose Nanofiber–Ag Nanoparticle Composite for High-Performance Humidity Sensor

**DOI:** 10.3390/nano14040343

**Published:** 2024-02-10

**Authors:** Mijin Won, Minhun Jung, Jaehwan Kim, Dong-Soo Kim

**Affiliations:** 1 Department of Creative Convergence Engineering, Hanbat National University, Yuseong-ku, Daejeon 34158, Republic of Korea; 2 Creative Research Center for Nanocellulose Future Composites, Inha University, Incheon 22212, Republic of Korea

**Keywords:** humidity sensor, cellulose nanofiber, silver nanoparticles, printing

## Abstract

This paper reports a high-performance humidity sensor made using a novel cellulose nanofiber (CNF)–silver nanoparticle (AgNP) sensing material. The interdigital electrode pattern was printed via reverse-offset printing using Ag nano-ink, and the sensing layer on the printed interdigitated electrode (IDE) was formed by depositing the CNF-AgNP composite via inkjet printing. The structure and morphology of the CNF-AgNP layer are characterized using ultraviolet–visible spectroscopy, an X-ray diffractometer, field emission scanning electron microscopy, energy-dispersive X-ray analysis, and transmission electron microscopy. The humidity-sensing performance of the prepared sensors is evaluated by measuring the impedance changes under the relative humidity variation between 10 and 90% relative humidity. The CNF-AgNP sensor exhibited very sensitive and fast humidity-sensing responses compared to the CNF sensor. The electrode distance effect and the response and recovery times are investigated. The enhanced humidity-sensing performance is reflected in the increased conductivity of the Ag nanoparticles and the adsorption of free water molecules associated with the porous characteristics of the CNF layer. The CNF-AgNP composite enables the development of highly sensitive, fast-responding, reproducible, flexible, and inexpensive humidity sensors.

## 1. Introduction

Humidity sensors play a critical role in our lives, with applications in various fields such as living environment monitoring, smart agriculture, smart logistics, packaging, and storage. The wide range of humidity sensor applications requires highly sensitive and fast-responding humidity sensors, and such demand has led to numerous efforts to develop new humidity sensors. Humidity sensors can be classified into different types based on the detection mechanism, including acoustic wave, capacitive, resistive, electrochemical, and quartz crystal microbalance humidity sensors [1,2,3,4,5]. Resistance- and impedance-type sensors belong to resistive types. Among these, the thin-film resistance humidity sensor has been widely researched owing to its ease of manufacture, low cost, and facile circuit integration [2,6,7]. This type of humidity sensor displays a signal associated with a change in impedance due to the interaction between the sensing material and water molecules.

Various functional materials such as cellulose, metal oxides, metal nanowires, porous ceramics, polymers, organic semiconductors, nanoclays, graphene, and carbon nanotubes have been used as humidity-sensing elements [8,9,10,11,12,13]. Cellulose has received significant attention because it is renewable and the most abundant raw material on earth, making its use environmentally sustainable. Cellulose is a colorless, odorless, and non-toxic solid polymer with desirable properties such as high mechanical strength, hydrophilicity, relative thermal stability, biocompatibility, low cost, and eco-friendliness [14]. In recent years, cellulose-based composites have been researched for coatings, pharmaceuticals, laminates, fibers, optical films, smart materials, and flexible sensing devices [15,16,17,18,19]. Recently, an all-cellulose-derived humidity sensor was made by using the direct laser writing of electrodes onto TEMPO-oxidized cellulose paper [18]. The TEMPO-oxidized cellulose paper with sodium carboxylate groups provides a satisfactory humidity-sensing performance and is converted to conductive and moisture-stable electrodes directly via laser-induced carbonization, demonstrating high sensitivity and linearity over a wide range of relative humidity.

This study investigated a highly sensitive and fast-responding humidity sensor using a 100% printing method by applying silver nanoparticles (AgNPs) to cellulose nanofiber (CNF). An interdigitated electrode (IDE) pattern was fabricated via the reverse-offset printing process, and the CNF-AgNP was used as the sensing material synthesized by formulating an ink with uniformly dispersed AgNPs from the reduction of CNF. Owing to the high conductivity of AgNPs and the porous properties of CNF, the CNF-AgNP sensing layer is innovated for high-performance humidity sensors. The sensing performance was tested between a 10% and 90% relative humidity (RH) range. The preparation and characterization of the humidity sensor are explained.

## 2. Experimental Methods

### 2.1. Materials

A 1.1 mm thick glass substrate was used as the substrate of the sensing material. The CNF solution was received from the Creative Research Center for Nanocellulose Future Composites, Inha University, where the CNF was isolated from hardwood pulp using TEMPO-oxidation and aqueous counter-collision methods [20]. AgNO_3_ (0.02 mol/L) and NaBH_4_ were purchased from Samchun Chemicals Co., Ltd. (Daegu, Korea) and Sigma-Aldrich (St. Louis, MO, USA), respectively. The Ag nano-ink (DGH 55-HTG) for reverse offset during electrode pattern printing was purchased from ANP Co., Ltd. (Sejong, Korea) All listed materials were used without additional purification steps.

### 2.2. Synthesis of CNF-AgNP Composite

**Figure 1:**
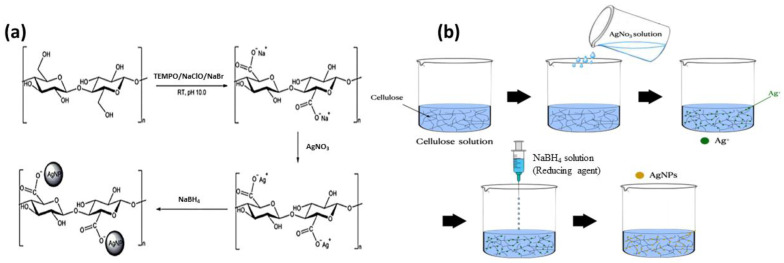
(**a**) Synthesis mechanism of CNF-NP composite and (**b**) Synthesis route of CNF-NP composite.

First, a dilute mixture was prepared by adding 2 g of distilled water to 1 g of CNF and stirring for 1 h. Then, 0.5 g of AgNO_3_ (0.02 mol/L) was added to the diluted CNF solution and mixed for 4 h, after which 1 mL of a mixture of 1 mL NaBH_4_ (0.1 M) and 9 mL distilled water was slowly added drop by drop and stirred for 24 h to prepare the CNF-AgNP solution. The synthesis mechanism and route of the CNF-AgNP composite are presented in Figure 1.

### 2.3. Characterization of CNF-AgNPs

The CNF and CNF-AgNP composite were characterized using ultraviolet–visible spectroscopy (UV–vis, Lambda 1050+, Perkin Elmer, Hong Kong, China), an X-ray diffractometer (XRD, XPERT-PRO, Bruker, Billerica, MA, USA), field emission scanning electron microscopy (FE-SEM, S-4000, Hitachi, Tokyo, Japan), energy-dispersive X-ray analysis (EDAX, Mahwah, NJ, USA), and transmission electron microscopy (TEM, CM200, Philips, Amsterdam, The Netherlands).

### 2.4. Humidity Sensor Fabrication

**Figure 2:**
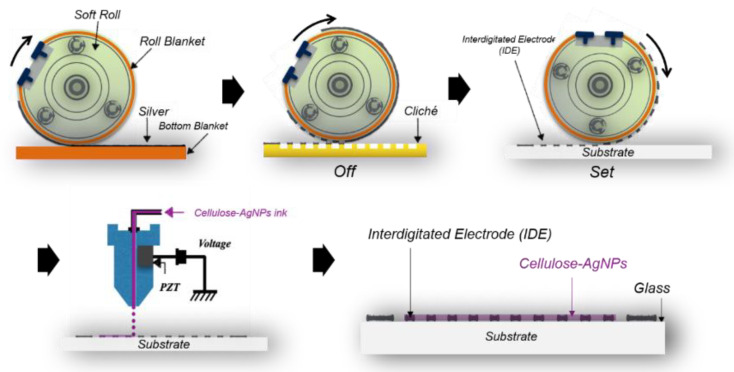
The fabrication process of the CNF-AgNP humidity sensor.

Figure 2 illustrates the schematic of the humidity sensor fabrication process. First, the IDE pattern was printed via reverse-offset printing using Ag nano-ink. The printed IDE pattern was then sintered in an oven at 400 °C for 20 min. The sensing layer on the printed IDE was formed by depositing the CNF-AgNP composite via inkjet printing, followed by heat treatment in an oven at 150 °C for 5 min. The same process was repeated for the CNF solution to prepare a CNF humidity sensor for comparison with the CNF-AgNP humidity sensor. The fabrication process is shown in Figure 2.

### 2.5. Performance Evaluation

**Figure 3:**
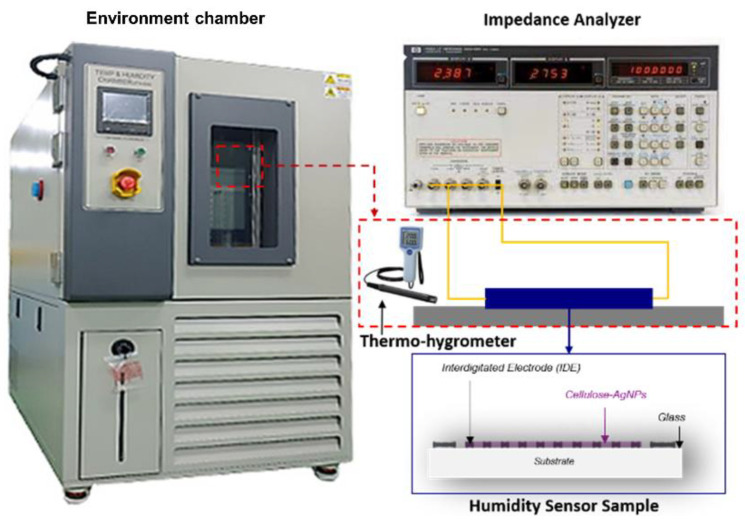
Performance test of CNF-AgNP humidity sensor.

To evaluate the performance of the fabricated CNF and CNF-AgNP humidity sensors, the impedance change of the sensors under the humidity change was measured using an impedance analyzer (Agilent 4192A, HP, Santa Clara, CA, USA) at 1 kHz and 1 V. The internal temperature and humidity were controlled using an environmental chamber (TEMP&HUMID CHAMBER, BSTECH Co., Ltd., Daejeon, Republic of Korea). To determine the accurate response characteristics of the sensors, the relative humidity (RH) was increased by 10%RH step from 10 to 90%RH. In addition, a commercial thermo-hygrometer (Sato, SK-110TRH type 4, Tokyo, Japan) was used to monitor the temperature and humidity inside the chamber. A schematic diagram of the measurement procedure is shown in Figure 3.

## 3. Results and Discussion

### 3.1. Characterization of the CNF-AgNP Composite

**Figure 4:**
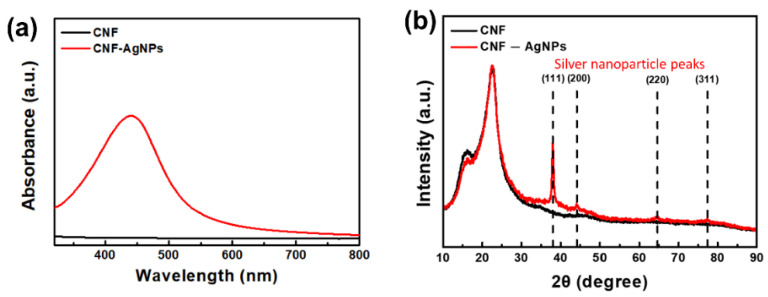
Optical characteristics of neat CNF film and CNF-AgNP composite film: (**a**) UV–vis absorption spectra and (**b**) XRD curves.

Figure 4a shows the UV–vis absorption spectra of the neat CNF film and the CNF-AgNP composite film, reflecting the successful formation of a AgNP. AgNPs formed from the redox reaction between the Ag^+^ ions of AgNO_3_ and NaBH_4_ were revealed from the cellulose spectrum of the characteristic UV–vis absorption band generally in the 400–450 nm range, as well as from the visible yellow color change [21,22,23]. This color persisted in the CNF-AgNP compound for 3 months, indicating that the AgNPs synthesized on cellulose exhibit excellent stability [24].

The presence of AgNPs was also confirmed using XRD. As shown in Figure 4b, diffraction peaks corresponding to the (111), (200), (220), and (311) planes of AgNPs appeared at 38°, 44°, 64°, and 77°, respectively [19]. In addition, the XRD peaks of CNF at 16°, 22°, and 34° were confirmed to be consistent with the (110), (200), and (004) planes of cellulose I [25].

**Figure 5:**
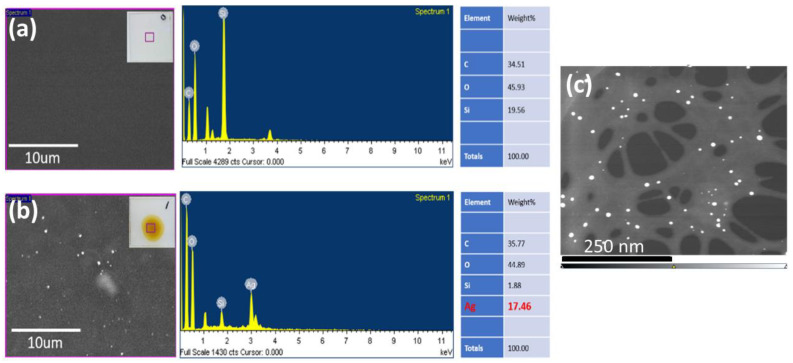
SEM images and EDX results of (**a**) neat CNF film and (**b**) CNF-AgNP composite film. (**c**) TEM of CNF-AgNP composite. White dots represent AgNPs.

As illustrated in Figure 1a, the formation of AgNPs on the CNF film was facilitated by its large surface area with abundant hydroxyl groups, resulting in stable and uniform dispersion of Ag^+^ charges. As the mobility of Ag^+^ decreases due to its interaction with the hydroxyl groups of the CNF, the growth of AgNPs is prevented, and stable formation of AgNPs on the surface of coarse CNF can be achieved [26]. To analyze the CNF-AgNPs, FE-SEM, EDAX, and TEM were employed. The CNF suspension and CNF-AgNP solution were diluted with distilled water and dropped onto a wafer substrate (Figure 5). Figure 5a,b show the SEM and EDX results of neat CNF and the CNF-AgNP composite, showing 17.46 wt% of the Ag element is present. Figure 5c shows the TEM image of the CNF-AgNP composite. AgNPs (white dots) attached to the surface of the CNF were shown to have a diameter of 10–20 nm, demonstrating the successful formation of the CNF-AgNP composite.

### 3.2. Fabrication of CNF-AgNPs Sensor

**Figure 6:**
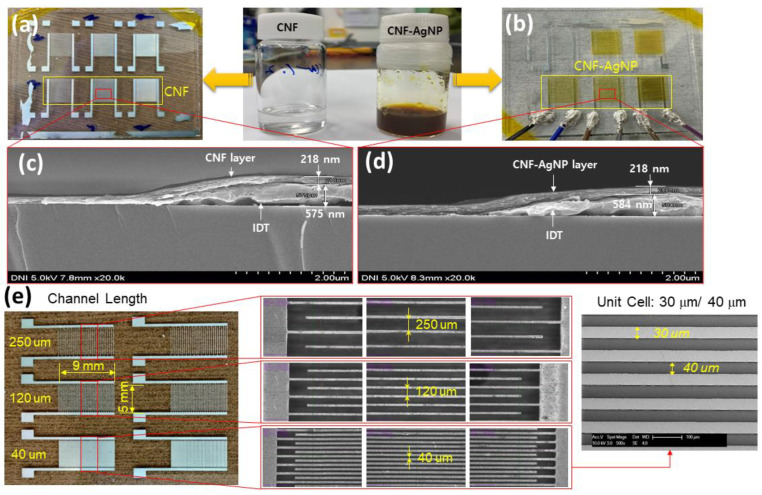
(**a**,**b**) Photograph of neat CNF and CNF-AgNP humidity sensors. (**c**,**d**) Cross-sectional SEM images of neat CNF and CNF-AgNP humidity sensors. (**e**) Photographs and SEM images of IDT patterns with different electrode distances: 40, 120, and 250 mm.

The CNF-AgNP sensor was fabricated using reverse-offset and inkjet printing, as illustrated in Figure 4. Reverse-offset printing was used for the precise patterning of IDEs to improve sensor accuracy. The IDEs patterned using reverse-offset printing had a line width of 30 µm, and multiple sets of electrodes were printed. Figure 6 shows the photographs and SEM images of the fabricated sensors. The number of electrodes was 100, 50, and 30, and the distance between electrodes was 40 µm, 120 µm, and 250 µm, respectively. Each set of IDEs was printed on a 1.1 mm thick glass substrate using a silver nano-ink via the reverse-offset process. Then, sensing layers were inkjet-printed using CNF and the developed CNF-AgNP composite inks. Finally, the humidity sensors were prepared by heat treatment of the printed sensing layers in an oven at 150 °C for 5 min for moisture removal. The line width and gap of the IDEs of the fabricated sensors were measured using an optical microscope.

### 3.3. Humidity Response

**Figure 7:**
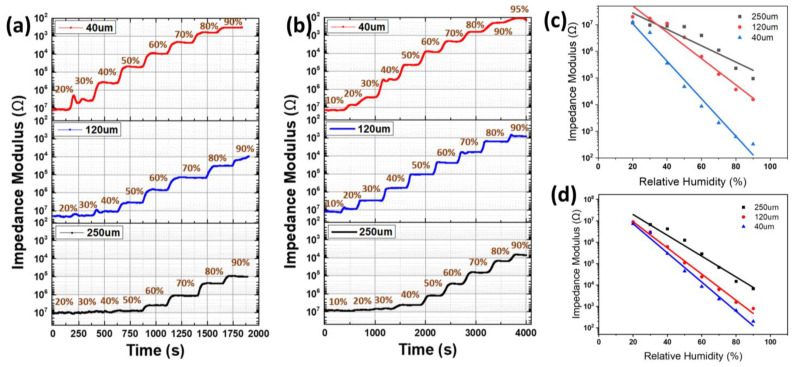
Humidity sensor responses with different electrode distances and RH levels: impedance curves with the time of (**a**) the CNF humidity sensor and (**b**) the CNF-AgNP sensor, and impedance curves with RH levels of (**c**) the CNF humidity sensor and (**d**) the CNF-AgNP sensor.

Figure 7a,b show the relative humidity–response curves of the CNF and CNF-AgNP humidity sensors concerning different electrode distances. As the RH level increases, the impedance values of the sensors hugely decrease. These huge impedance changes are promising for sensing humidity changes. The humidity-sensing performance enhanced as the number of electrodes varied from 30 to 50 to 100 (i.e., electrode spacing decreased from 250 µm to 120 µm to 40 µm). Figure 7c,d show the impedance response to a change in relative humidity. Linear fitting to the CNF and CNF-AgNP sensors yielded coefficients of determination of 0.9926 and 0.985, respectively; the normalized root-mean-square error of CNF and CNF-AgNPs was 2.51% and 3.16%, respectively. The CNF-AgNP sensors exhibit a superior sensing response than the CNF sensors, close to a linear response. The linear response is clear as the electrode distance decreased to 40 µm. The experimental results also demonstrate that the CNF-AgNP sensor can sense a broad range of humidity levels, between a low 10%RH and a high 90%RH. The sensitivity of the CNF-AgNP humidity sensor (electrode distance = 40 μm) is 0.0654 (log∆R/%RH). It is nearly twice as sensitive as the all-cellulose-derived humidity sensor made by using the direct laser writing of electrodes onto TEMPO-oxidized cellulose paper [18]. Table 1 shows a comparison of their performance.

It is postulated that increasing the number of electrodes reduces the sensors’ impedance by increasing the ion mobility between the electrodes and the polymer matrix that forms the humidity-sensing layer. As the humidity increases, the conductivity of the CNF layer is affected because the CNF layer swells and absorbs free water molecules that can activate ions and migrate inside the humidity-sensing layer [27,28]. Moreover, the sensitivity of the CNF-AgNP sensor is superior to the CNF sensor, as demonstrated in Figure 7a,b. This result is also consistent with the fact that, as relative humidity increases, water molecules facilitate electron transfer via hydrophilic functional groups such as hydroxyl groups on the CNF surface and AgNPs to reduce resistance. The Maxwell–Wagner–Sillars (MWS) polarization process states that polymer–filler interfacial interactions induce changes in the dielectric properties of composite materials. In the CNF-AgNP composite, the large interfacial area serves as multiple sites for enhanced MWS effect [29]. The reduction in resistance can be explained by the increase in charge at the interface under various relaxation times (*t* = ε/σ, where ε is permittivity and σ is conductivity) when a current flows across the interface of two dielectric materials. Thus, the superior humidity-sensing performance of the CNF-AgNP sensor to that of the neat CNF sensor is valid according to the MWS effect. Such enhanced humidity-sensing performance is associated with the increased conductivity of the Ag nanoparticles and the adsorption of free water molecules due to the excellent porous characteristics of the CNF layer.

**Figure 8:**
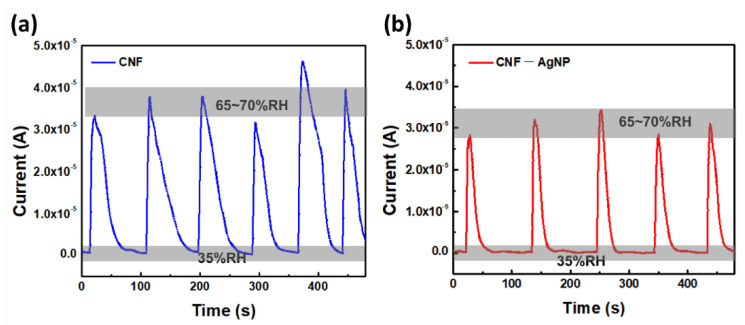
Time responses of the fabricated humidity sensors when the RH level changed between 35% and 65~70%RH repeatedly: (**a**) the CNF sensor and (**b**) the CNF-AgNP sensor.

Figure 8 shows the time responses of the CNF and CNF-AgNP printed sensors under different %RH. The response time is the rising time from 35%RH to 65–70%RH, and the recovery time is the falling time from 65–70%RH to 35%RH. At varying 35–70%RH levels, the response times of the CNF and CNF-AgNP sensors were 7 s and 4 s, and the recovery times were 75 s and 34 s, respectively, indicating the fast humidity-sensing performance of the sensor containing AgNPs. The longer recovery time than response time is attributed to free water molecule accumulation in the CNF layer. The composite sensor showed a fast response time of 4 s and a recovery time of 34 s. These times are much faster than the all-cellulose-derived humidity sensor (60 s/495 s) [18].

## 4. Conclusions

A highly sensitive and fast-responding humidity sensor was developed using a novel CNF-AgNP sensing material with the reverse-offset and inkjet printing method. The structure and morphology of the CNF-AgNP layer were characterized using UV–vis, XRD, SEM, EDX, and TEM, indicating the excellent dispersion of Ag nanoparticles in the CNF matrix. The humidity-sensing performance of the prepared sensors was evaluated by measuring the impedance changes under a relative humidity variation of between 10 and 90%RH. The CNF-AgNP sensor exhibited a more sensitive humidity-sensing performance than the CNF sensor. As the electrode distance decreased, the humidity-sensing performance enhanced. In addition, in the 35–70%RH variation, the CNF-AgNP sensor showed fast sensing responses: the response and recovery times were 4 s and 43 s, respectively. The high-performance humidity sensing of the CNF-AgNP sensor is associated with the increased conductivity of the Ag nanoparticles and the adsorption of free water molecules due to the excellent porous characteristics of the CNF layer. The CNF-AgNP composite offers advantages that enable the development of highly sensitive, fast-responding, reproducible, flexible, and inexpensive humidity sensors. The developed humidity sensor is expected to have various applications in flexible and wearable electronic devices.

## Figures and Tables

**Table 1:** Performance comparison of CNF, CNF-AgNP, and TEMPO-cellulose humidity sensors.

Name	Humidity Range (%RH)	Response (Ω)	$\mathrm{Sensitivity}\;(\frac{log\Delta R}{\%RH})$	Response/Recovery Time (s)	Reference
CNF	10~90	1 × 10^7^~3 × 10^2^	0.0565	7/75	This work
CNF-AgNP	10~90	1.7 × 10^7^~1 × 10^2^	0.0654	4/34	
TEMPO-cellulose	11~98	9 × 10^8^~8 × 10^5^	0.0351	60/495	[18]

## Data Availability

Data will be provided upon request.
